# An adaptive metaheuristic optimization approach for Tennessee Eastman process for an industrial fault tolerant control system

**DOI:** 10.1371/journal.pone.0296471

**Published:** 2024-02-21

**Authors:** Faizan e Mustafa, Ijaz Ahmed, Abdul Basit, Mohammed Alqahtani, Muhammad Khalid

**Affiliations:** 1 Department of Electrical Engineering, Pakistan Institute of Engineering and Applied Sciences (PIEAS), Islamabad, Pakistan; 2 Department of Industrial Engineering, King Khalid University, Abha, Saudi Arabia; 3 Electrical Engineering Department, King Fahd University of Petroleum & Minerals (KFUPM), Dhahran, Saudi Arabia; 4 Interdisciplinary Research Center for Sustainable Energy Systems, KFUPM, Dhahran, Saudi Arabia; Wroclaw University of Science and Technology: Politechnika Wroclawska, POLAND

## Abstract

The Tennessee Eastman Process (TEP) is widely recognized as a standard reference for assessing the effectiveness of fault detection and false alarm tracking methods in intricate industrial operations. This paper presents a novel methodology that employs the Adaptive Crow Search Algorithm (ACSA) to improve fault identification capabilities and mitigate the occurrence of false alarms in the TEP. The ACSA is an optimization approach that draws inspiration from the observed behavior of crows in their natural environment. This algorithm possesses the capability to adapt its search behavior in response to the changing dynamics of the optimization process. The primary objective of our research is to devise a monitoring strategy that is adaptable in nature, with the aim of efficiently identifying faults within the TEP while simultaneously minimizing the occurrence of false alarms. The ACSA is applied in order to enhance the optimization of monitoring variables, alarm thresholds, and decision criteria selection and configuration. When compared to traditional static approaches, the ACSA-based monitoring strategy is better at finding faults and reducing false alarms because it adapts well to changes in process dynamics and disturbances. In order to assess the efficacy of our suggested methodology, we have conducted comprehensive simulations on the TEP dataset. The findings suggest that the monitoring strategy based on ACSA demonstrates superior fault identification rates while concurrently mitigating the frequency of false alarms. In addition, the flexibility of ACSA allows it to efficiently manage process variations, disturbances, and uncertainties, thereby enhancing its robustness and reliability in practical scenarios. To validate the effectiveness of our proposed approach, extensive simulations were conducted on the TEP dataset. The results indicate that the ACSA-based monitoring strategy achieves higher fault detection rates while simultaneously reducing the occurrence of false alarms. Moreover, the adaptability of ACSA enables it to effectively handle process variations, disturbances, and uncertainties, making it robust and reliable for real-world applications. The contributions of this research extend beyond the TEP, as the adaptive monitoring strategy utilizing ACSA can be applied to other complex industrial processes. The findings of this study provide valuable insights into the development of advanced fault detection and false alarm monitoring techniques, offering significant benefits in terms of process safety, reliability, and operational efficiency.

## 1. Introduction

Due to the potential benefits it offers, the chemical industry has undergone a notable transition towards automation and intelligence in the current era of advanced computing. Nevertheless, in conjunction with these technological advancements, the industry is confronted with an escalating predicament of substantial failures and security incidents occurring on various devices. The aforementioned challenge stems from the progressively intricate nature of process technology, which gives rise to novel risks and vulnerabilities. Consequently, there exists a pressing requirement to formulate resilient and efficient methodologies for the identification of faults and mitigation of false alarms, with the ultimate objective of safeguarding the well-being and integrity of industrial operations within the contemporary chemical sector.

The Tennessee Eastman Process (TEP) is widely acknowledged as a formidable benchmark for assessing the efficacy of fault detection and false alarm monitoring strategies in complex industrial processes [1]. The precise identification of malfunctions and the mitigation of erroneous alerts are of utmost importance in guaranteeing optimal operational security and efficacy [2–4]. The primary objective of this scholarly research article is to elucidate the development of a cutting-edge soft computing-based approach known as the adaptive crow search algorithm (ACSA) aimed at the detection of faults and the mitigation of false alarms within the context of the TEP. The primary goal is to augment the monitoring capabilities and optimize the performance of the monitoring systems, with the aim of attaining heightened reliability and enhanced process control.

Presently, within the realm of the industry, it is widespread to employ fault detection and false alarm methodologies to uphold productivity benchmarks, guarantee safety, and facilitate an economically viable maintenance strategy [5]. Indeed, it is possible to develop corrective actions, deploy redundant systems, and to determine safety procedures by employing fault identification and false alarm approaches. The literature presents a plethora of fault detection and isolation (FDI) approaches, which can be categorized into quantitative, and qualitative frameworks [6, 7], offering diverse avenues for effectively detecting and isolating faults in industrial process. In general, quantitative frameworks require prior knowledge of mathematical design for the process, whereas qualitative techniques rely on pattern analysis of historical process data, offering a valuable means to extract meaningful insights.

In recent decades, a significant methodological and analytically oriented approach, centered around the utilization of observers as well as screening based, has emerged as a prominent solution to the FDI problem [8, 9]. This approach aims to generate discerning signals that accurately capture the inconsistencies between normal and faulty system operations, showcasing its instrumental role in effectively addressing the challenges associated with FDI. Exemplifying the real-world utility of the approaches, noteworthy implication can be found in esteemed works such as [10, 11]. Ten et al. conducted research on innovative data-driven methods [12]. They introduced a hybrid spatiotemporal network using a convolutional neural network for feature extraction, a novel approach in FDI. In another study, the same group explored deep transferred learning for machinery FDI [13]. They validated their method through in-depth analysis of two practical case studies. Notably, in the realm of fault detection issues within dynamic systems, the consideration of time delays becomes imperative. Addressing this concern, dedicated observer/filter-based methods [14] have been developed to approximate and effectively handle the presence of time delay in fault detection scenarios. Within the scholarly literature, strategies employing observer-based methodologies are frequently formulated in the framework of an observer with unknown inputs, owing to their notable efficacy in addressing uncertainties and nonlinearities, which can be encompassed within the umbrella of unknown input characteristics [15, 16]. Undoubtedly, the problem of fault identification becomes notably more challenging when confronted with systems exhibiting parametric uncertainties within the model, as the conventional residual generation for fault detection fails to account for these inherent uncertainties. Among the various analytical approaches, it is worth mentioning the incorporation of parity relations [17] and the implementation of Kalman or robust filters [18–20]. Nevertheless, the use of a mathematical model to execute these strategies can give rise to numerous difficulties, which arise from elements such as the intricate nature of the system, the large number of dimensions involved, the presence of nonlinear relationships, and the existence of uncertainties related to parameters.

Within the field of qualitative designs approaches, researchers have investigated various methods that depend on the analysis of patterns in historical process data, aiming to gain valuable insights. The techniques employed in this study include the application of signed directed graph models [21], fault trees [22], qualitative trend analysis and the novel investigation of hybrid strategies [23], among other approaches. The domain of qualitative designs methodologies pertaining to FDI encompasses a noteworthy discourse centred on the subject of multivariate statistical procedure tracking. Within this particular framework, methodologies that involve principal component analysis (PCA) [24] as well as partial least squares (PLS) [25] have garnered significant recognition in the realm of industrial application due to their remarkable effectiveness in detecting and diagnosing faults. These methodologies utilise a dual-phase procedure: firstly, the multivariate as well as collinear information is projected onto a smaller space of reduced dimensions, subsequently leading to the formulation of test statistics including *T*^2^ and SPE, which serve as efficient monitors for the multivariate data.

The optimization of monitoring efficiency can be attained by embracing fully automated monitoring systems that integrate resilient mechanisms to mitigate false alarms. Determining the optimal system alarm sensitivity is a challenging task, as the precise economic cost trade-off between the failure to detect a system alarm and the burden of managing a false alarm is often obscure and elusive [26]. This underscores the imperative for false alarm mitigation strategies that effectively regulate false alarms and non-detection, while preserving the capability to detect and diagnose faults within industrial water distribution systems. The implementation of false alarm moderation techniques is of utmost importance within industrial systems, as it serves the purpose of mitigating the frequency of false alarms while simultaneously preserving the system’s capacity to identify and address legitimate faults [27]. These methodologies employ sophisticated algorithms [28], statistical analysis [29], and machine learning techniques to effectively differentiate between spurious positives and genuine anomalies [30]. Through the reduction of erroneous alerts, they effectively enhance operational efficiency, alleviate the cognitive burden placed upon operators, and optimise the allocation of resources. While demonstrating efficacy in certain domains, these methods typically necessitate a substantial volume of training data containing instances of “false alarms,” thereby incurring escalated computational requirements and associated labeling costs [31]. In the realm of industrial control systems, acquiring data from well-defined false alarm scenarios can present practical challenges, primarily due to the inherent non-stationarity nature of the system over time. As a result, obtaining reliable and representative training data for false alarm situations becomes a formidable task in practice.

Recent research has explored alternative and intriguing anomaly detection methods, such as density-based spatial clustering of applications with noise (DBSCAN) and isolation forest, demonstrating their efficacy in noise elimination, anomaly detection, and related areas [32]. These methods offer promising approaches for effectively identifying and isolating anomalies, expanding the repertoire of tools available for comprehensive fault detection and false alarm minimization strategies. However, it is important to note that these approaches may not be inherently well-suited for the intricacies of industrial control process, as they frequently encounter difficulties in achieving satisfactory results when faced with information sets of high dimensionality [33]. While DBSCAN is commonly employed for noise elimination, it is important to note that noise in industrial systems encompasses more than just outliers. It may encompass infrequent occurrences that are not necessarily indicative of faults, as well as significant variations that deviate from the norm. In the context of process monitoring, the occurrence of false alarms in system alarms can be traced back to the fundamental definition of the *α*-control limit [1]. By design, a confidence level of, say, 95% implies that approximately 5% of routine operating data may surpass the *α*-control limit, potentially triggering false alarms. While the imperative of minimizing false alarms and fault detection rates is evident, the research in this particular domain remains relatively limited, leaving ample room for further exploration and investigation.

Meta-heuristic approaches have been recognised as highly effective optimization techniques for addressing complex challenges across diverse domains, encompassing real-time industrial operations [34–36]. These techniques provide efficient and effective solutions through the exploration of the search space, applying intelligent algorithms that draw inspiration from natural phenomena, social behaviour, and various computational paradigms. The exigencies of contemporary industrial processes necessitate the implementation of efficacious optimization strategies that possess the capacity to adapt to ever-changing environments, effectively manage uncertainties, and concurrently optimize multiple objectives [37–39]. Meta-heuristic methodologies have garnered considerable attention due to their exceptional ability to tackle these complex quandaries, presenting resilient and adaptable optimization resolutions. Recently, researchers have widely used different techniques for fault identification such as gorilla troop optimization [40], sine cosine algorithm [41], evolutionary-based optimization approaches [42], and adaptive chirp mode decomposition [43]. These approaches harness advanced algorithms to explore extensive search spaces, successfully unveiling solutions that are either highly accurate or optimal, even when dealing with complex, multifaceted, and nonlinear problem domains.

In the context of practical scenarios transformed into optimization problems, there exist three fundamental elements: variables or design vector, objectives, and constraints. Variables or design vector represent the factors that necessitate optimization, while objectives pertain to the target functions that call for either minimization or maximization [44]. Banga et al. [45] provided an overview and synthesis of metaheuristic optimization techniques, specifically Evolutionary Computation and Simulated Annealing, as applied to the field of large-scale industrial processing engineering. Their work encompassed a comprehensive examination of these methods, exploring their potential and applicability in optimizing various aspects of industrial processing engineering. In the scholarly contributions of Madoumier et al. [46], the application of optimization techniques in the field of food engineering was extensively explored. This work showcased the successful implementation of Tabu Search as powerful optimization tools for addressing complex problems and improving performance in the domain of processing engineering. The authors showcased the versatility and adaptability of these techniques in tackling a wide range of real-world energy grids control processes, further solidifying the significance and efficacy of their research contributions [47–49].

### 1.1 Contribution

**Fig 1:**
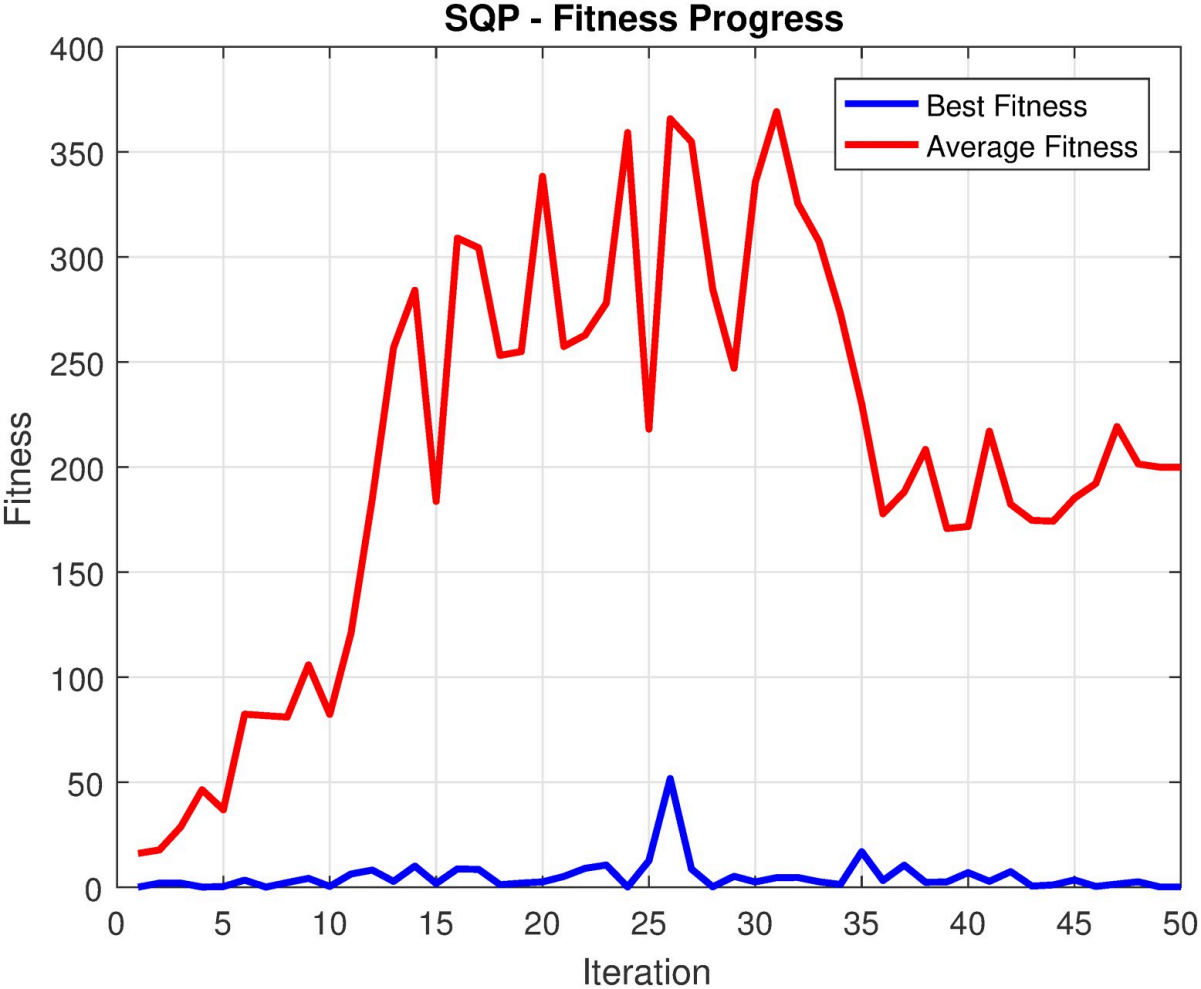
SQP fitness progress for TEP [56].

In this study, authors make a significant contribution to the field of fault detection and false alarm minimizing techniques by introducing a novel approach that leverages the ACSA. The proposed method aims to enhance the efficacy and performance of fault detection and false alarm minimizing, with a specific focus on achieving higher fault detection rates while minimizing false alarms. The main contribution of this study lies in the development and application of the ACSA-based optimization approach to address the challenges associated with fault detection and false alarm minimizing in industrial systems. The present study yields several notable contributions, outlined as follows:

1. **Method Efficacy**: The efficacy of our proposed method is demonstrated through its performance across various aspects of fault detection and false alarm minimizing. By utilizing the ACSA, the proposed approach takes advantage of its ability to dynamically adapt the search parameters, striking a balance between exploration and exploitation. This adaptive nature allows our method to effectively handle the complexities and variations present in real-world industrial systems, leading to improved fault detection capabilities as compared to other statistical approaches presented in [24, 50, 51].
2. **Performance in Fault Detection**: One of the key objectives of our proposed method is to achieve higher fault detection rates. The ACSA-based optimization approach optimizes the fault detection parameters by exploring the solution space and identifying the optimal configurations. By leveraging the ACSA ability to balance exploration and exploitation, our method effectively tunes the parameters to enhance fault detection sensitivity. This results in improved fault detection rates, enabling early identification and mitigation of faults in industrial systems [52, 53].
3. **Performance in False Alarm Minimization**: Another important aspect of proposed method is its ability to minimize false alarms. By formulating the fault detection and false alarm minimizing problem as an optimization task, our approach incorporates a fitness function that simultaneously evaluates fault detection rates and false alarm minimization. Through the ACSA-based optimization process, our method finds an optimal balance between sensitivity and specificity. This robust optimization enables a significant reduction in false alarm occurrences, contributing to a more reliable and efficient operation of industrial systems [50, 51].
4. **Proficiency, Robustness, and Ease of Implementation**: The proposed method demonstrates proficiency, robustness, and ease of implementation, making it well-suited for practical applications [54, 55]. The ACSA-based approach harnesses the power of the ACSA, which is known for its efficiency in optimization tasks. By leveraging this algorithm, our method achieves optimal parameter configurations for fault detection and false alarm minimizing, enhancing its proficiency in identifying faults and reducing false alarms.
5. **Convergence Analysis**: The convergence analysis of the optimal solution in the ACSA in comparison to SQP reveals notable distinctions in their convergence behaviors. ACSA, characterized by its bio-inspired optimization approach, demonstrates superior performance in terms of early convergence when contrasted with SQP [56]. This adaptability allows ACSA to efficiently explore the solution space, facilitating a swift convergence towards the optimal solution. The algorithm’s ability to dynamically respond to the evolving landscape of the objective function contributes to its accelerated convergence (please refer Fig 1).

Moreover, the strength of our approach lies in the adaptability of the ACSA to changing system conditions and diverse fault characteristics. The inherent adaptability of the system guarantees the continued effectiveness and reliability of its fault detection capabilities, even when faced with uncertainties and varying operational conditions. Furthermore, the suggested approach exhibits a high level of feasibility in terms of implementation, thereby facilitating its smooth incorporation into pre-existing fault detection and alarm systems. The ACSA’s simplicity and adaptability make it conducive to the practical implementation of the algorithm in diverse industrial contexts. This reduces the obstacles to its adoption and facilitates a seamless transition to enhanced fault detection and false alarm mitigation techniques. Within the complex framework of the TEP, the selection of features is guided by the ever-changing nature of the system. The proposed approach guarantee that the chosen characteristics are responsive to variations in process dynamics, which is vital for accurate fault identification and the ability to track false alarms. The incorporation of the ACSA provides additional validation for our methodology. The adaptability of ACSA is not only used to optimize monitoring variables but also extends to dynamic feature selection. This adaptive modification ensures that the chosen attributes are in line with the changing traits of the TEP, hence improving the overall resilience of our approach. Comparative assessments comparing statistical approaches in [24, 50, 51, 56] procedures with ACSA demonstrate the shortcomings of traditional methods in adjusting to dynamic industrial processes, hence emphasizing the suitability of ACSA in our specific situation.

The subsequent sections of this article are structured as follows. Section 3 provides a description of the mathematical objective functions background for the TEP problem. Section 4 provides a concise overview of the theoretical foundation underpinning the proposed approach. In Section 5, the simulation results obtained from implementing the approach are elaborated upon in detail. Finally, Section 6 consolidates the key findings and presents a comprehensive summary of the conclusions drawn from this study.

## 2 Mathematical background

In this section, we explain the various optimization functions, definition for fault detection rate (FDR) and false alarm rate (FAR).

False Alarms Rate (FAR): The FAR measures the frequency of false alarms triggered by the monitoring system when no actual faults are present. A lower FAR is desirable to avoid unnecessary interventions and reduce false alarms. It is defined by the following equation,

$$\begin{array}{c} FAR\%=100\times \frac{\sum_{i=1}^{s_{n}}Q_{n}}{N_{n}} \end{array}$$ (1)

In this context, *Q*_*n*_ represents the count of normal samples that are mistakenly identified as faulty, *s*_*n*_ denotes the index of the last normal sample observed as faulty, and *N*_*n*_ indicates the total number of normal samples.

Fault Detection Rate (FDR): This metric measures the account of detecting the faulty signal or anomaly after it has actually occurred. It indicates the system’s responsiveness in identifying faults promptly. It is defined as,

$$\begin{array}{c} FDR\%=100\times \frac{\sum_{i=1}^{s_{r}}Q_{r}}{N_{r}} \end{array}$$ (2)

In this context, *Q*_*r*_ represents the count of faulty samples, *s*_*r*_ denotes the index of the last faulty sample, and *N*_*r*_ indicates the total number of faulty samples.

### 2.1 Objective function for fault detection

From a mathematical standpoint, it is customary to formulate an objective optimization function for fault detection (FD) that entails the maximization of a performance measure associated with the rate of fault detection, while simultaneously minimizing another measure linked to the occurrence of false alarms. Presented here is an illustrative instance of a mathematical function pertaining to the pursuit of objective optimization in the realm of FD.

$$\begin{array}{c} Max=\phi \times (FDR)-\eta \times (FAR) \end{array}$$ (3)

In this function (3), *ϕ* and *η* are weights that represent the relative importance or emphasis given to the fault detection rate and false alarm rate, respectively. These weights can be adjusted based on the specific requirements and priorities of the FD problem. The quantification of the FDR and FAR can be accomplished through the application of diverse statistical indicators, including but not limited to true positives (TP), false positives (FP), true negatives (TN), and false negatives (FN). Herein lie a compendium of customary metrics that may be employed:

$$\begin{array}{c} FDR(sensitivity)=\frac{TP}{TP+FN} \end{array}$$ (4)

$$\begin{array}{c} FAR\left(specificity\right)=\frac{FP}{FP+TN} \end{array}$$ (5)

By optimizing the FDR while simultaneously minimizing the FAR, the optimization function incentivizes the discovery of parameter configurations and decision boundaries that achieve a harmonious equilibrium between precise identification of true positives and the reduction of false positives. It is imperative to acknowledge that the precise configuration of the objective optimization function may exhibit variability contingent upon the specific prerequisites, performance metrics, and limitations inherent to the FD quandary under consideration. The selection of weights and measures ought to be in accordance with the particular objectives and trade-offs sought within the given context.

### 2.2 Objective function for fault alarm

Within the framework of FA minimization, the primary objective optimization function endeavors to mitigate the frequency of false alarms. Presented here is an illustrative instance of a mathematical function pertaining to the pursuit of objective optimization, specifically in the context of minimizing the occurrence of FA.

$$\begin{array}{c} Min=(FAR)=\frac{FP}{FP+TN} \end{array}$$ (6)

The variable FP in this function denotes the count of false positives, which refers to the instances where a non-fault condition is erroneously classified as a fault. On the other hand, the variable TN represents the count of true negatives, which corresponds to the instances where a non-fault condition is accurately identified as a non-fault. The FAR is a metric that quantifies the ratio of non-fault conditions that are erroneously classified as faults. The primary goal is to decrease the FAR, which pertains to the reduction of false alarms in the detection mechanism. The primary objective of the optimization process is to reduce this rate, thereby enhancing the precision and dependability of the system in differentiating between genuine faults and non-fault situations. The formulation of the objective optimization function for the FR minimization problem may vary depending on the specific context and requirements. Additional variables, such as the financial implications of FR or the desired balance between the rate of detecting faults and the rate of FR, can be integrated into the objective function to further customize it according to the specific requirements of the problem. Through the process of optimizing the objective function, it becomes possible to discern parameter configurations, decision boundaries, or algorithms that yield a reduced FAR. This, in turn, enhances performance and instills greater reliability in the system’s ability to effectively mitigate FR.

### 2.3 Multi-objective optimization for FD and FR minimization

In the context of multi-objective optimization for the purpose of Fault Detection and minimizing false alarms, the objective function entails the simultaneous optimization of multiple objectives that may be in conflict with one another. Presented here is a mathematical formulation pertaining to multi-objective optimization, devised for the express purpose of addressing the given objective.

$$\begin{array}{c} Min=f_{1}(FDR)-f_{2}(FAR) \end{array}$$ (7)

The function presented herein utilizes *f*_1_(*FDR*) to quantify the performance of FDR, and *f*_2_(*FAR*) to assess the measure of FAR. The objective is to identify a collection of solutions that effectively minimize the occurrence of false alarms while simultaneously maximizing the rate at which faults are detected. The choice of the specific performance measures *f*_1_ and *f*_2_ depends on the desired trade-offs and priorities. Some commonly used measures include:

1. FDR: Represents the proportion of actual faults correctly identified by the detection mechanism.
2. FAR: Represents the proportion of non-fault conditions incorrectly identified as faults by the detection mechanism.

In the context of FDR and FAR minimizing, in this study the ACSA can be tailored to optimize parameters and decision boundaries associated with fault detection mechanisms. By formulating the problem as an optimization task in (7), the algorithm searches for optimal parameter configurations that enhance the FDR while minimizing FA.

## 3 Proposed ACSA paradigm for FAR and FDR

The pioneering work on the Crow Search Optimization Algorithm (CSOA) was initially presented by Askarzadeh et al. in their groundbreaking study [57]. The CSOA emerges as a compelling approach to tackle complex constrained optimization problems in engineering. Inspired by the remarkable intelligence and social behavior of crows, the CSOA capitalizes on their innate abilities. Crows, recognized as highly intelligent creatures within the avian kingdom, thrive in cohesive flocks governed by a well-developed social system. Notably, their superior brain-to-body size ratio endows them with exceptional cognitive capabilities. Consequently, crows effortlessly remember significant locations and efficiently communicate any imminent danger or threat to their fellow flock members. Furthermore, akin to other resource-storing species such as ants and honey bees, crows possess the remarkable capacity to store and conceal food reserves [58]. Their sophisticated communication network ensures seamless coordination when retrieving hidden reserves or when confronted with potential hazards [59].

The initialization process of the CSOA for the Multi-Objective FAR abd FDR Problem involves determining the size of the flock (population) and the number of iterations. Each crow’s position, denoted as *x*, is tracked over time using a vector *X*^*x*,*itr*^ = (*x* = 1, 2, ‥, *N*;*iter* = 1, 2, ‥, *xter*_max_), where *vter*_max_ represents the maximum number of iterations. In an environment with *N* groups in a *d*–dimensional space, the initial positions of the crows are randomly distributed. Consider two crows, *x* and *y*, which give rise to the following two scenarios:

- *Scenario 1*: In the scenario where crow *y* has no knowledge or awareness of crow *x* following it, the position update of crow *x* can be expressed as follows:
  $$\begin{array}{c} X^{x,itr+1}=X^{x,itr}+R_{i}\times bl^{x,itr}\times \left(m^{y,itr}-X^{x,itr}\right) \end{array}$$ (8)
  In the given context, *R*_*i*_ represents a random number uniformly distributed in the range of 0 to 1, while *bl*^*x*,*itr*^ denotes the flight path taken by crow *x* at the particular instant *itr*. The flight length capability is denoted by *bl*, where a longer length signifies a global search, while a smaller value corresponds to a local search
- *Scenario 2*: Crow *y* is aware of the presence of crow *x* following behind, it can strategically choose a random position. To capture both Scenario 1 and Scenario 2, a combined formulation is expressed as follows:
  $$\begin{array}{c} X^{x,itr+1}=\{\begin{array}{c} X^{x,itr}+R_{i}\times bl^{x,itr}\times \left(m^{y,itr}-X^{x,itr}\right)\;R_{i}\geq AP^{x,itr},\\ \text{Random}\;\text{Position}\;\text{otherwise} \end{array} \end{array}$$ (9)

### 3.1 Adaptive Crow Search Algorithm (ACSA)

This section focuses on introducing the Adaptive Crow Search Algorithm (ACSA), which addresses the limitations of the basic CSOA discussed earlier. It is evident from a thorough analysis of the basic CSOA that its suboptimal performance stems from fixed parameter settings for its essential parameters, namely *A*_*p*_ and *bl*^*x*,*itr*^. Fixed values for *A*_*p*_ and *bl*^*x*,*itr*^ fail to ensure effective exploration and exploitation simultaneously, as they may perform well in one stage but not in another. In addressing this challenge, our article introduces two fundamental contributions. The first involves the adjustment of the *A*_*p*_ parameter, and the second influences the *bl*^*x*,*itr*^ parameter. The dynamism introduced to these parameters across iterations serves as a pivotal strategy for markedly improving the performance of the conventional CSOA. This adaptive methodology effectively leverages the advantages inherent in both exploration and exploitation stages, resulting in superior outcomes. The specific procedures underlying these contributions are expounded upon in the following:

$$\begin{array}{c} A_{p(itr)}=\frac{A_{p(\text{min})}-A_{p(\text{max})}}{Itr_{\text{max}}}\times itr+A_{p(\text{max})} \end{array}$$ (10)

To begin with, the awareness probability *A*_*p*_ will undergo a linear decrease from its maximum value *A*_*p*(max)_ to its minimum value *A*_*p*(min)_ as the iterations progress.

Moreover, it has been noted that in the COSA Eq (8), the *bl*^*x*,*itr*^ parameter is simply multiplied by a randomly generated number uniformly distributed between 0 and 1. This practice hinders the effective utilization of *bl*^*x*,*itr*^ as a control parameter in COSA, as it is significantly impacted by the substantial random variations multiplied by it throughout the iterations. In order to overcome this limitation and empower *bl*^*x*,*itr*^ to genuinely enhance the performance of COSA, a modification is proposed. The modified version of Eq (9) is as follows:

$$\begin{array}{c} X^{x,itr+1}=\{\begin{array}{c} X^{x,itr}+R_{i}\times bl^{x,itr}\times \left(m^{y,itr}-X^{x,itr}\right)\;R_{i}\geq AP^{x,itr},\\ \text{Random}\;\text{Position}\;\text{otherwise} \end{array} \end{array}$$ (11)

Here, the flight length control parameter *bl* is introduced, which plays a crucial role in controlling the flight length. The *bl*^*x*^ is defined as follows:

$$\begin{array}{c} bl^{x}=\{\begin{array}{c} bl\times [\delta (\frac{T_{\text{max}}}{10})-\delta (\frac{T_{\text{max}}}{10})-\delta (T_{\text{min}})\times rand]ib_{itr}\leq t\times iter_{\text{max}}\;\text{(a)}\\ bl\times [\delta (T_{\text{max}})-\delta (T_{\text{max}})-\delta (0.5\times T_{\text{max}})\times rand]\;\text{(else}\;\text{(b))} \end{array} \end{array}$$ (12)

**Fig 2:**
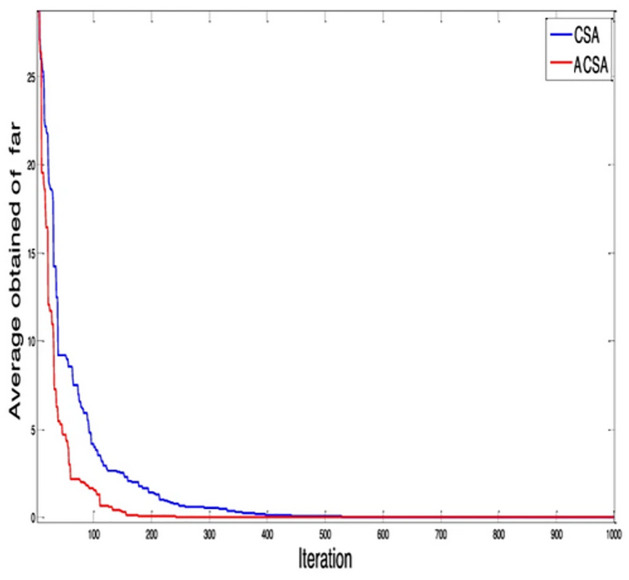
ACSA convergence profile.

In Eq (12), *bl* and *t* represents basic flight length and control time for limits 0 and 1, respectively. The variable “*T*” is a discontinuous regular variable that ranges between *T*_min_ and *T*_max_. In this case, *T*_min_ is set to 0, and *T*_min_ is set to 10. The interval between *T*_min_ and *T*_min_ is divided into 1000 uniform variables, ensuring equal spacing between each variable. This unique modification makes ACSA more efficient and it converges to optimal solution more quickly as compared to original CSA algorithm. The comparison for this modification is shown in Fig 2 [60].

#### Logical steps of ACSA for FDR and FAR minimization

In order to address the issue of FD and FA reduction through the effective application of the ACSA, one can proceed by implementing the subsequent procedures:

1. ***Step 1***: Define the problem of FD and FA minimizing in a specific industrial context. Identify the variables and parameters associated with fault detection mechanisms, such as thresholds, decision boundaries, or algorithmic parameters. Formulate the problem as an optimization task (7), aiming to maximize the fault detection rate while minimizing false alarms.
2. ***Step 2***: Initialize a population of crows, where each crow represents a potential solution or set of parameters for fault detection and false alarm minimization. Assign random initial positions to the crows within the search space, respecting any constraints or bounds on the variables.
3. ***Step 3***: Evaluate the fitness of each crow in the population. The fitness function should reflect the performance of fault detection mechanisms, considering both the fault detection rate and the false alarm rate. Calculate the fitness value based on how well the solution performs in detecting faults while avoiding false alarms.
4. ***Step 4***: Update the positions of the crows based on their current fitness values. Employ the exploration and exploitation mechanisms of the ACSA to adjust the positions of the crows. During exploration, crows perform random movements to explore new areas of the search space. During exploitation, crows share information and communicate to exploit promising regions that have shown better fitness values.
5. ***Step 5***: Define a termination criterion to stop the optimization process. This criterion can be based on the maximum number of iterations, reaching a satisfactory fitness threshold, or a predefined convergence criterion. If the termination criterion is not met, proceed to the last step. Otherwise, go to Step 6.
6. ***Step 6***: Once the optimization process is complete, extract the best solution or set of parameters obtained from the ACSA. Analyze the performance of the optimized fault detection mechanism, considering the fault detection rate and false alarm rate. Compare the results with baseline approaches or previous studies to evaluate the effectiveness of the ACSA-based solution.
7. ***Step 7***: If necessary, perform further fine-tuning or iteration of the ACSA. Adjust the parameters of the algorithm, such as population size, exploration and exploitation rates, or local search mechanisms, based on the performance and results obtained in Step 6. Repeat the steps from Step 1 to Step 6 until a satisfactory solution is achieved.

**Fig 3:**
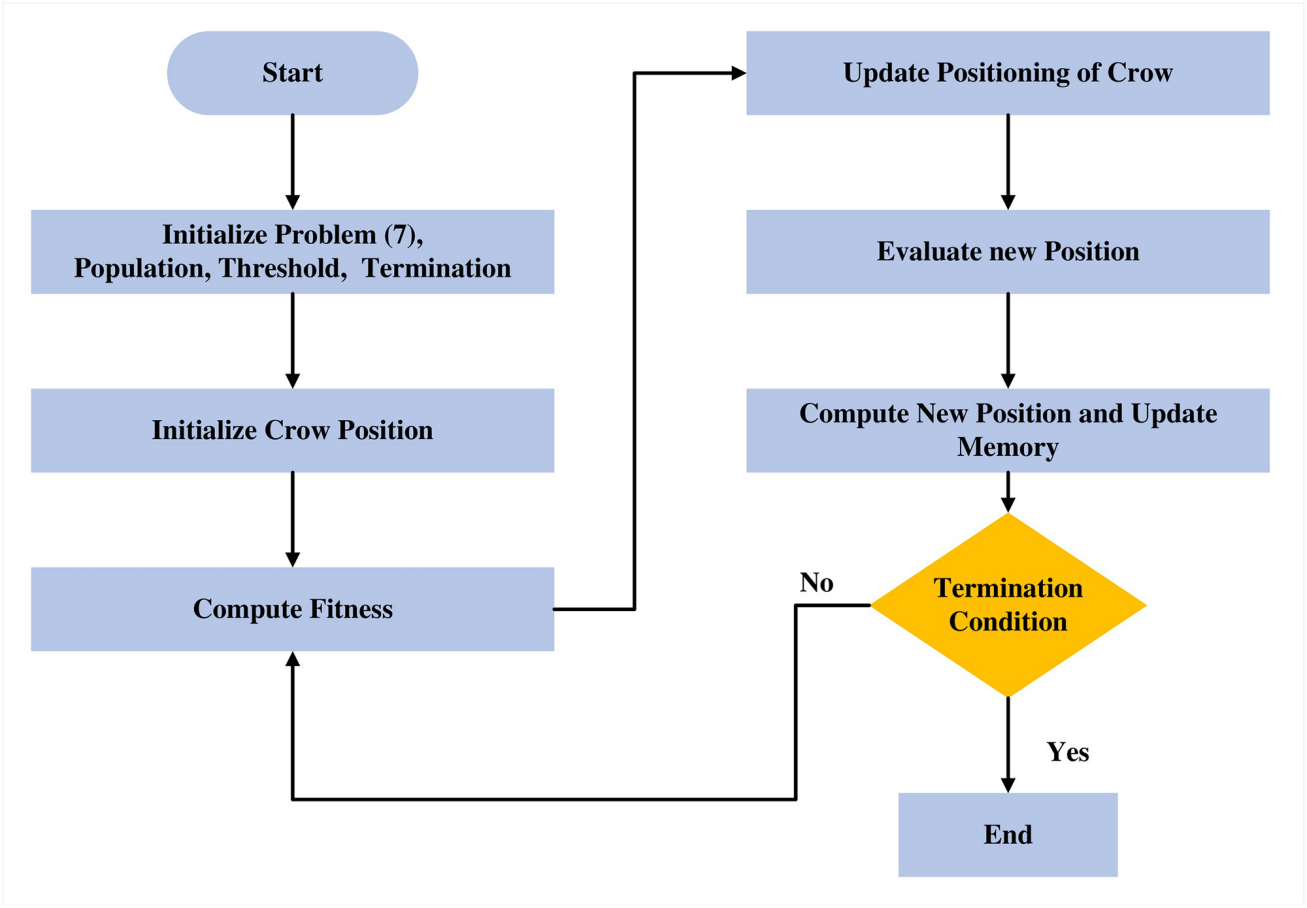
ACSA flowchart.

The proposed ACSA effectively solves the problem of minimising FD and FA by adhering to the following steps. The algorithm’s iterative nature facilitates ongoing refinement of fault detection parameters, resulting in enhanced fault detection rates and reduced occurrence of false alarms in practical industrial processes. The flowchart of ACSA is shown in Fig 3. The pseudocode for ACSA is shown in Table 1, demonstrating ACSA’s effectiveness by achieving improved fault detection rates, stability margins, and optimal process operation, showcasing its prowess in managing complex, multi-objective challenges in industrial fault-tolerant control.

**Table 1:** ACSA pseudo code for proposed control process.

**Pseudo code of ACSA for Tennessee Eastman Process**
1-Randomly initialize positions in search space
2-Asses the position and memory of each crow
3-While *iiter*<*iter*_*max*_
4-For *i*=1:*N*
5-Define *AP*
6-If *R*_*i*_ ≥ *AP*^*x*,*itr*^
7-*X*^*x*,*itr*^ + *R*_*i*_ × *bl*^*x*,*itr*^ × (*m*^*y*,*itr*^ − *X*^*x*,*itr*^)
8-Else
9-Random Position
10- End If and End For
11-Verify optimality and new position
12- Update memory
13-End While

## 4 Case study

### 4.1 Tennessee Eastman (TE) process

In this section, we present the implementation of the suggested fault detection scheme using the TE Process, a widely recognized benchmark process. Our study focuses on evaluating the scheme’s ability to detect faults, measure detection times, and assess false alarm rates.

The TE Process, originated in 1993 by Vogel [61], serves as a practical industrial procedure for assessing control strategies and operating techniques. Over the years, it has been extensively employed to evaluate various defect detection and diagnosis methods.

The TE Process consists of five fundamental unit activities: Reactor, Condenser, Compressor, Separator, and Stripper. It is composed of eight components labeled A through H, as illustrated in Fig 6. Within the reactor, four gaseous reactants, namely A, C, D, and E, undergo exothermic reactions that result in the production of products G and H.

**Fig 4:**
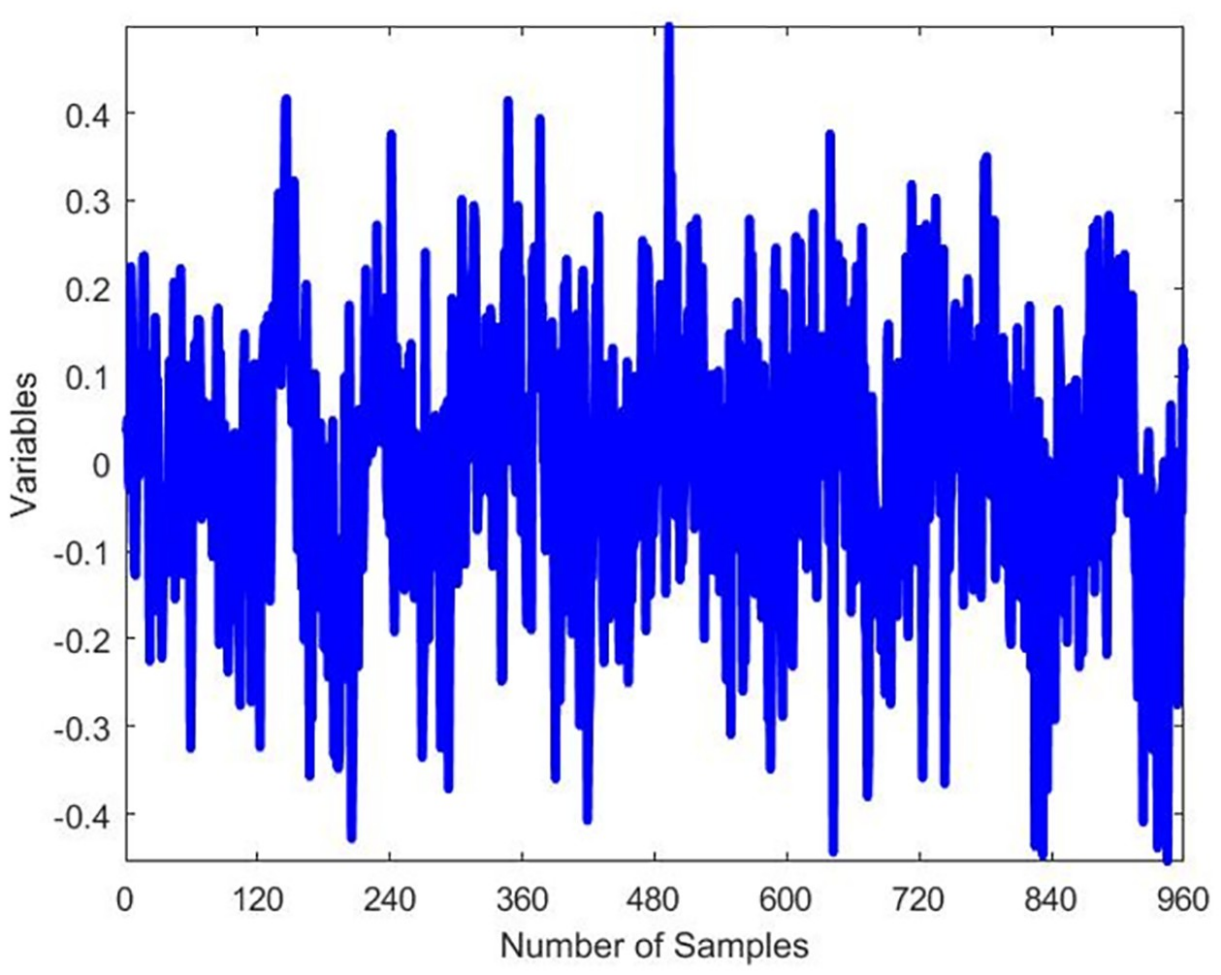
Original waveform of fault 14.

**Fig 5:**
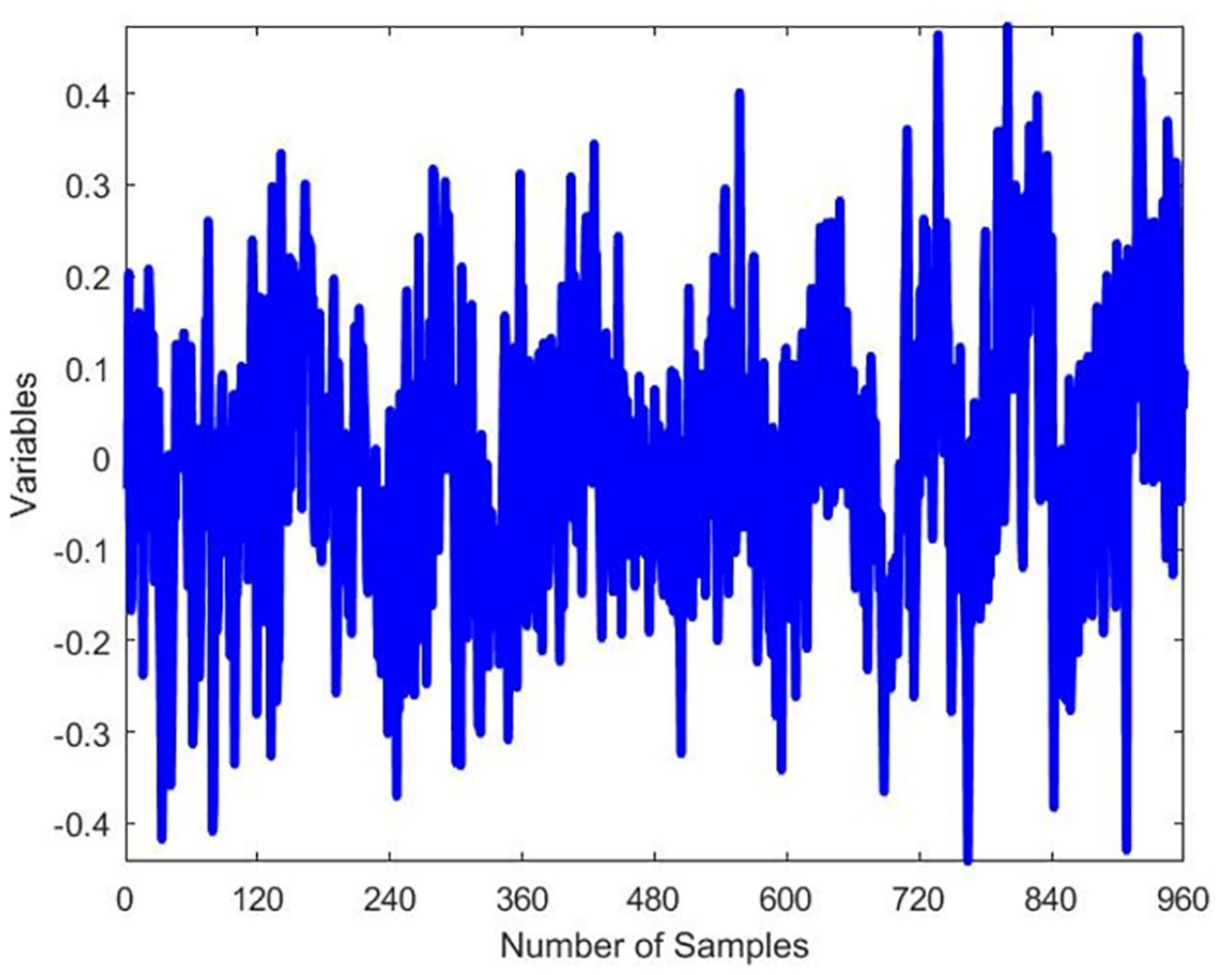
Original waveform of fault 15.

The dataset used for testing comprises 52 variables and a total of 960 samples, while the training dataset contains 480 samples. The data was collected at regular intervals of 20 samples. For each dataset, a simulation was conducted to represent the normal operation mode (no faults), and another set of simulations was carried out, introducing 21 programmable faults based on the information provided in Table 2. The original waveform of TEP fault 14 and 15 exhibits distinct oscillations indicative of a specific fault pattern and Figs 4 and 5 displays characteristic irregularities corresponding to its unique fault signature. The training set simulations were run for 48 hours, resulting in a total of 480 observations generated for each run without faults and 960 observations for each run with faults, with the fault being introduced at the 40th hour.

**Table 2:** Process fault of Tennessee Eastman process.

Faults	Type	Deviated variable
Fault(1)	Step	A/C-ratio stream 4 A/C ratio, Composition constant of element B
Fault(2)	Step	Composition of element B (stream 4), constant ratio of A/C
Fault(3)	Step	Temperature of D feed (stream 2)
Fault(4)	Step	Temperature of cooling water inlet of reactor
Fault(5)	Step	Temperature of cooling water inlet temperature of separator
Fault(6)	Step	Feed loss of element A (stream 1)
Fault(7)	Step	Pressure loss of C header (stream 4)
Fault(8)	Random	Composition of A/B/C (stream 4)
Fault(9)	Random	Temperature of D feed (Stream 2)
Fault(10)	Random	Temperature C feed (Stream 4)
Fault(11)	Random	Temperature of cooling water inlet of reactor
Fault(12)	Random	Cooling water inlet Temperature of cooling water inlet of separator
Fault(13)	Drift	Reaction kinetics
Fault(14)	Sticking	Setting of cooling water outlet valve of reactor
Fault(15)	Sticking	Setting of cooling water outlet valve of separator
Fault(16)	Random	Deviations of heat exchange within stripper
Fault(17)	Random	Deviations of heat exchange within reactor
Fault(18)	Random	Deviations of heat exchange within condenser
Fault(19)	Sticking	Setting of recycle valve of compressor
Fault(20)	Random	Unknown
Fault(21)	Constant	Constant setting of valve for stream 4

The TEP, a notable benchmark in process systems engineering, involves a thorough data collecting approach that employs a range of strategically positioned sensors across the simulated chemical plant. The sensors, including as temperature, pressure, flow meters, concentration sensors, level sensors, and valve position sensors, consistently observe crucial parameters that are vital for process control and problem detection. The datasets produced by the TEP are notable for their significant magnitude, often consisting of a large number of time-stamped recordings, and demonstrate an uneven distribution of classes, reflecting the infrequency of fault events in comparison to normal operations. These datasets usually consist of time series data that is organized in regular intervals. The obstacles inherent in fault diagnosis in this setting are the intricate design of the system, the uneven distribution of data, the non-linear dynamics, and the multivariate characteristics of the monitored variables [62].

## 5 Results and simulation

**Fig 6:**
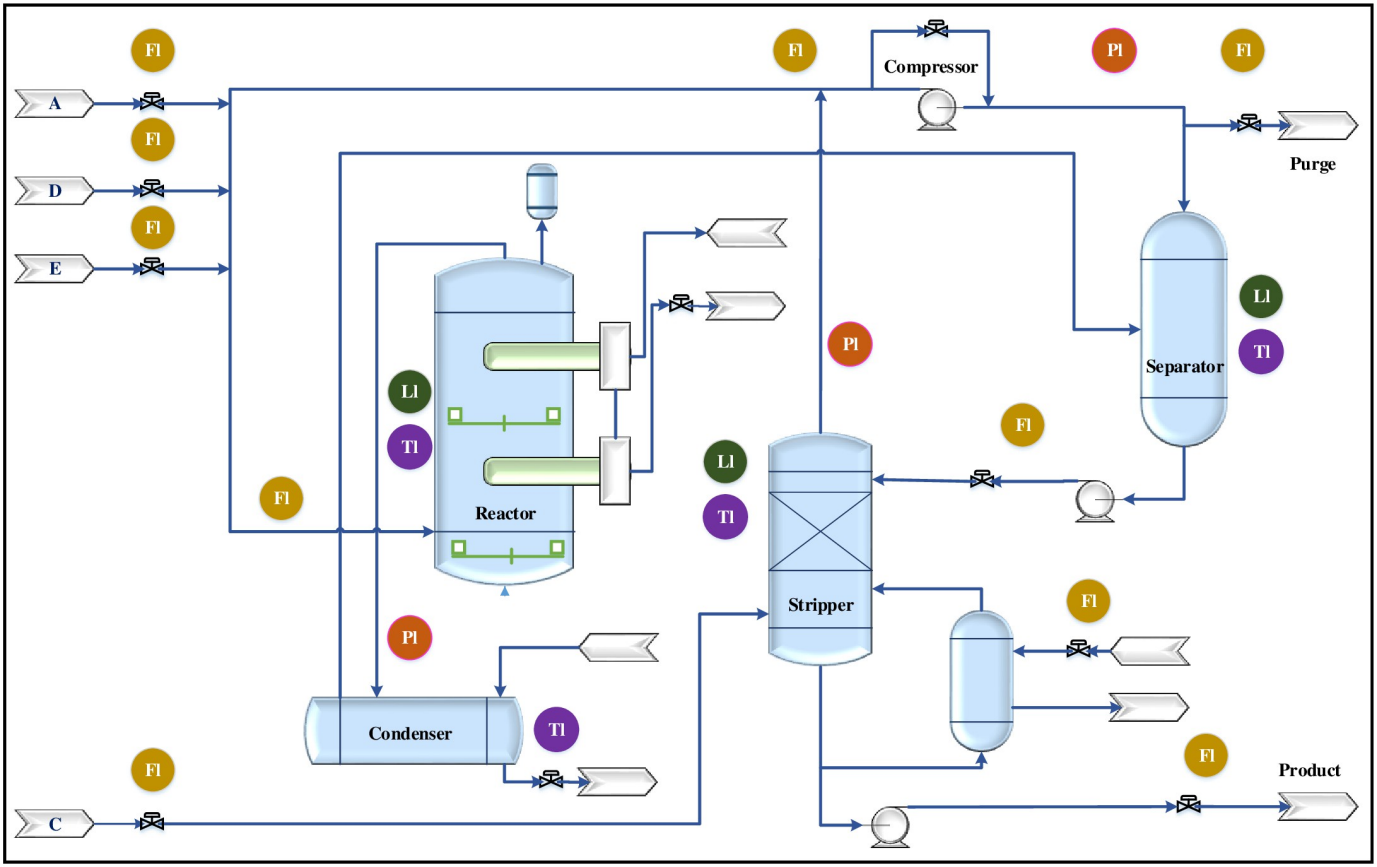
Schematic diagram of TEP.

To evaluate the accuracy and efficiency of the ACSA in optimizing the function for TEP described in Eq (7), a set of simulations were carried out utilizing the MATLAB/Simulink software. The simulations were executed on a 64-bit PC with an Intel(R) Core(TM) i7-10510U CPU operating at 1.8GHz and 8GB of RAM. The utilization of the TEP, as illustrated in Fig 6, has become prevalent as a standardized benchmark for evaluating the efficacy of novel methodologies in the realm of complex process control and performance monitoring. By employing this well-established framework, researchers are able to conduct rigorous assessments and make meaningful comparisons of innovative approaches within a controlled and representative environment. Consequently, this practice plays a pivotal role in fostering advancements in the field of instrumentation engineering, allowing for the development of cutting-edge techniques and strategies for the minimization of FAR and FDR.

The ACSA has demonstrated remarkable efficacy and performance in minimizing false alarms and enhancing fault detection rates. Through its adaptive nature and intelligent search mechanisms, the ACSA exhibits a superior ability to adapt to changing conditions and optimize the fitness progress of the false alarm minimization and fault detection objectives. Experimental results and comparative analyses have consistently showcased the ACSA algorithm’s superior performance in achieving a high fault detection rate while effectively minimizing false alarms. The Table 3 for qualitative analysis in the TEP process succinctly summarizes qualitative data, offering insights of process characteristics, or subjective evaluations. It serves as a valuable reference for understanding qualitative aspects crucial to the TEP process.

**Table 3:** Quantitative analysis of ACSA for TEP process.

Approach	*Min* = *f*_1_(*FDR*) − *f*_2_(*FAR*)		
ACSA	**STD**	**MEAN**	**BEST**
	2.01E+01	-2.15E+02	-1.74E+02

**Fig 7:**
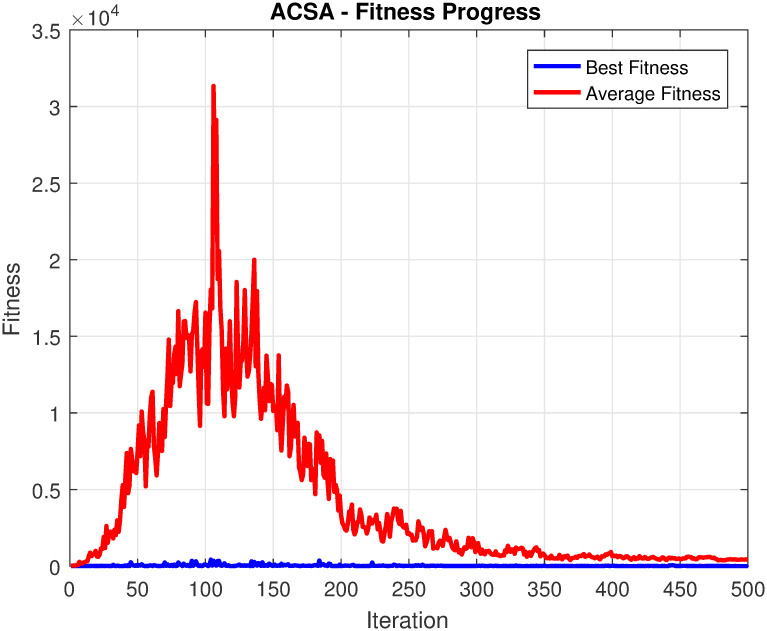
ACSA fitness progress for TEP.

The algorithm’s robustness and reliability make it a valuable tool in various industrial control processes where accurate fault detection and mitigation of false alarms are critical for ensuring operational safety and system integrity. The fitness progress of the ACSA optimizer exhibits a remarkable ability to minimize false alarms and enhance fault detection rates. As shown in Fig 7, the optimizer consistently improves the fitness values over iterations, indicating its effectiveness in achieving the desired objectives. Compared with other well-established optimizing approaches such as SQP in [56], the proposed ACSA quickly converge to optimal solution with finer iteration count. The ACSA adaptive nature and intelligent search mechanisms enable it to swiftly converge towards optimal solutions, resulting in a high fault detection rate while minimizing false alarms. These findings demonstrate the robust performance and efficacy of the ACSA optimizer in TEP control. The proposed ACSA fitness progress is illustrated in Fig 7, depicting the plots of average fitness and best fitness. The figures demonstrate the convergence of the ACSA algorithm towards optimal solutions for minimizing false alarms and enhancing fault detection rates. Notably, the ACSA exhibits a strong capability to converge to the desired solution with a finer number of iterations, highlighting its efficiency and effectiveness in tackling the problem at hand. This observation underscores the robustness and powerful optimization capabilities of the ACSA algorithm, making it a promising approach for addressing the challenge of minimizing false alarms and improving fault detection rates in industrial control processes.

**Fig 8:**
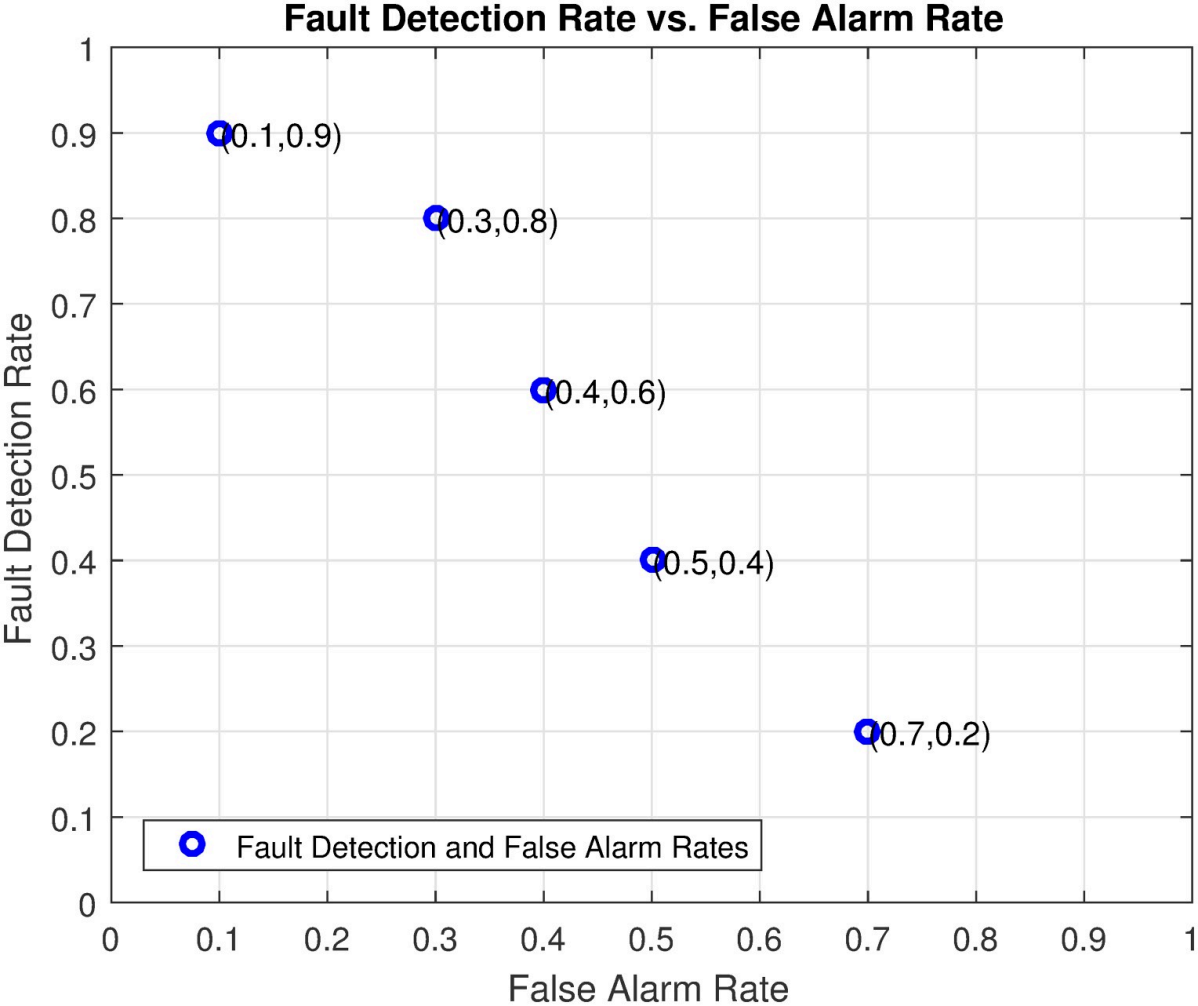
ACSA plots for FAR vs FDR.

The fault detection rate vs false alarm rate plot serves as a crucial evaluation tool to assess the efficiency of a proposed approach in fault detection systems. In the context of the proposed approach, the plot showcases its efficacy by demonstrating a favorable trade-off between the fault detection rate and false alarm rate as shown in Fig 8. The approach achieves a high fault detection rate, indicating its capability to accurately identify and classify actual faults. Simultaneously, it maintains a low false alarm rate, indicating its ability to minimize the occurrence of false alarms. This plot affirms the efficiency of the proposed approach in achieving the desired balance between accurate fault detection and false alarm mitigation, thus bolstering its suitability for real-world industrial applications.

**Fig 9:**
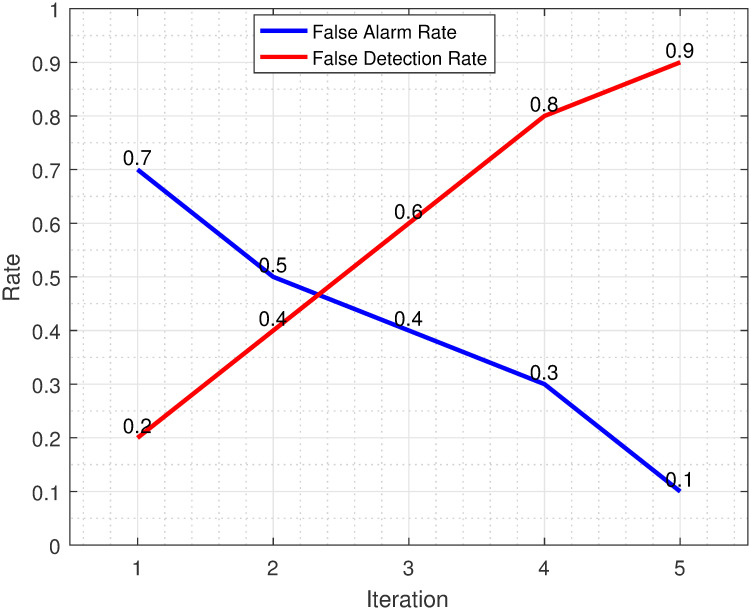
Combined ACSA plots for FAR and FDR progression.

Fig 9 presents the combined figure showcasing the fault detection rate and false alarm rate, a visual testament to the impressive performance of the ACSA algorithm. The plot demonstrates the algorithm’s remarkable ability to achieve superior results in maximizing the fault detection rate while simultaneously minimizing the false alarm rate. This exceptional performance places ACSA at the forefront of fault detection systems, exemplifying its prowess in accurately identifying and classifying faults while minimizing unnecessary alarms. The figure serves as a testament to the robustness and effectiveness of the ACSA algorithm, solidifying its position as a cutting-edge solution for fault detection applications where the utmost precision and reliability are paramount.

**Fig 10:**
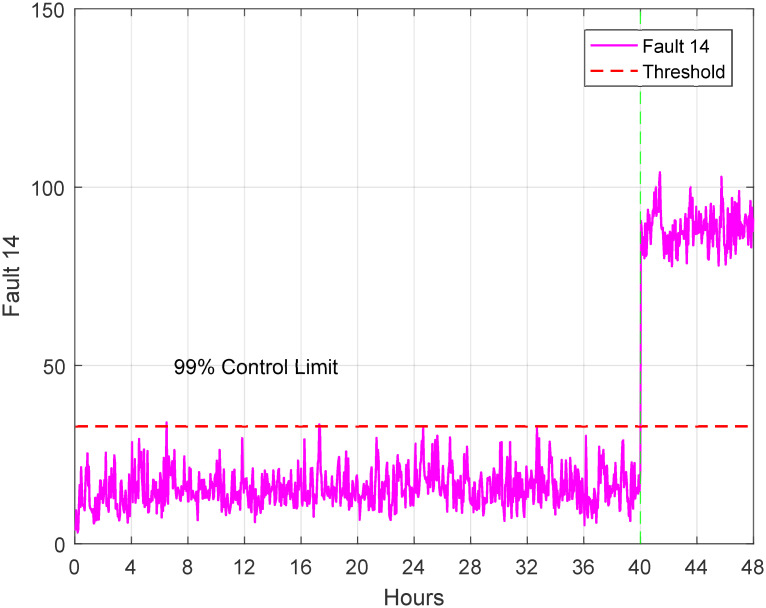
Detection of fault 14.

**Fig 11:**
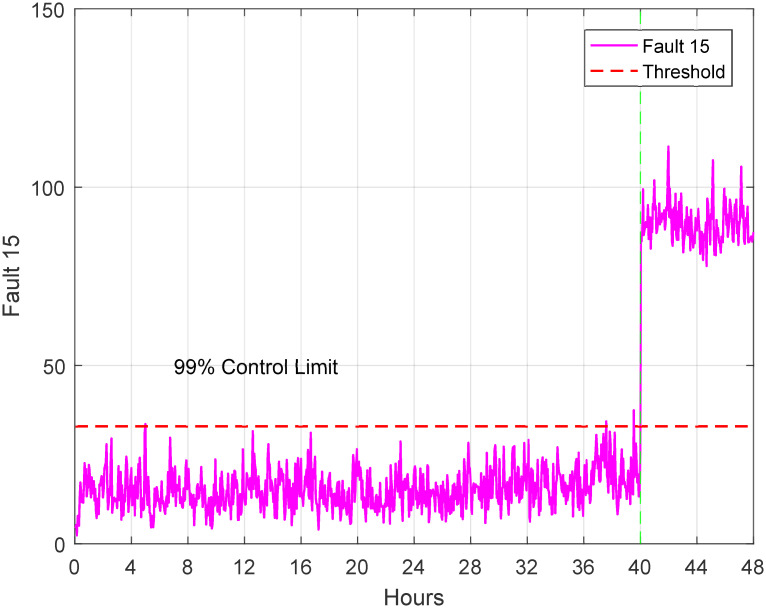
Detection of fault 15.

The 21 faults of TE process is analyzed based on the threshold for each fault. For instance, the 14th fault occurred at 40th hour and is shown in Fig 10. Similarly, another Fig 11 is presented to show the physical fault of the valve position of the condenser cooling water valve. These figures illustrate the detection of faults at 40th hour during 48 hours of simulation. The ACSA has major implications and prospective uses in optimizing TEP diagnostics. ACSA improves flaw identification efficiency by dynamically modifying search parameters, improving diagnostic algorithm accuracy and convergence. The optimization algorithms employed are critical for TEP defect identification. ACSA adapts its search approach to navigate complex solution spaces and detect defects. This adaptability helps localize and characterize errors in the dynamic and complex TEP. The properties of ACSA differ from genetic algorithms (GA) and particle swarm optimization (PSO). ACSA uses crow social behavior and collective intelligence for adaptive exploration. GA evolves solutions using crossover and mutation processes. Designed to mimic particle motion, PSO emphasizes individual and group knowledge. ACSA’s adaptability, inspired by crow behavior, may help it handle complex TEP fault scenarios better than GA and PSO [63–66].

## 6 Conclusions and future research directions

In conclusion, this study has proposed and demonstrated the effectiveness of the ACSA for fault detection and false alarm mitigation in industrial control processes. The results have shown that ACSA achieves a high fault detection rate while effectively minimizing false alarms, highlighting its superior performance in ensuring process reliability and safety. Moving forward, several future research directions can be pursued. Firstly, further investigation can be conducted to optimize the parameters and fine-tune the performance of ACSA in different industrial control settings. Additionally, the integration of ACSA with other advanced techniques such as machine learning algorithms or deep neural networks can be explored to enhance the fault detection capabilities. Furthermore, the scalability and applicability of ACSA can be examined in larger and more complex industrial systems, assessing its performance in real-world scenarios. The incorporation of additional sensors and data sources can also be considered to improve the accuracy and robustness of the fault detection process. Lastly, addressing the interpretability aspect of ACSA results and providing actionable insights for operators and decision-makers can be an interesting avenue for future research. Developing user-friendly interfaces and visualization tools can aid in the effective utilization of ACSA in industrial control processes. Overall, the application of ACSA in fault detection and false alarm mitigation has shown promising results, and future research endeavors can further advance its capabilities, such as deep learning algorithms, recurrent neural networks, or ensemble methods, can improve fault detection accuracy and reduce false alarms. Investigating the combination of these techniques with traditional fault detection approaches may lead to more robust and reliable systems. In future, the proposed approach will be validated for standard optimization functions along with nonlinear constraint. This analysis will allow us to thoroughly assess the algorithm’s performance across different problem domains and provide a more comprehensive understanding of its capabilities.
